# The interplay of reactive oxygen species and the epidermal growth factor receptor in tumor progression and drug resistance

**DOI:** 10.1186/s13046-018-0728-0

**Published:** 2018-03-16

**Authors:** Meng-Shih Weng, Jer-Hwa Chang, Wen-Yueh Hung, Yi-Chieh Yang, Ming-Hsien Chien

**Affiliations:** 1 Department of Nutritional Science, Fu Jen Catholic University, New Taipei City, Taiwan; 2 Department of Internal Medicine, School of Medicine, College of Medicine, Taipei Medical University, Taipei, Taiwan; 3 Division of Pulmonary Medicine, Department of Internal Medicine, Wan Fang Hospital, Taipei Medical University, Taipei, Taiwan; 4 Graduate Institute of Clinical Medicine, College of Medicine, Taipei Medical University, 250 Wu-Hsing Street, Taipei, 11031 Taiwan; 5 Genomics Research Center, Academia Sinica, Taipei, Taiwan; 6 Department of Medical Education and Research, Wan Fang Hospital, Taipei Medical University, Taipei, Taiwan

**Keywords:** Epidermal growth factor receptor, Reactive oxygen species, NADPH oxidase, Oxidation, Tumor progression, Drug resistance

## Abstract

**Background:**

The epidermal growth factor receptor (EGFR) plays important roles in cell survival, growth, differentiation, and tumorigenesis. Dysregulation of the EGFR is a common mechanism in cancer progression especially in non-small cell lung cancer (NSCLC).

**Main body:**

Suppression of the EGFR-mediated signaling pathway is used in cancer treatment. Furthermore, reactive oxygen species (ROS)-induced oxidative stress from mitochondrial dysfunction or NADPH oxidase (NOX) overactivation and ectopic expression of antioxidative enzymes were also indicated to be involved in EGFR-mediated tumor progression (proliferation, differentiation, migration, and invasion) and drug resistance (EGFR tyrosine kinase inhibitor (TKI)). The products of NOX, superoxide and hydrogen peroxide, are considered to be major types of ROS. ROS are not only toxic materials to cells but also signaling regulators of tumor progression. Oxidation of both the EGFR and downstream phosphatases by ROS enhances EGFR-mediated signaling and promotes tumor progression. This review primarily focuses on the recent literature with respect to the roles of the EGFR and ROS and correlations between ROS and the EGFR in tumor progression and EGFR TKI resistance.

**Short conclusion:**

The evidence discussed in this article can serve as a basis for basic and clinical research to understand how to modulate ROS levels to control the development and drug resistance of cancers.

## Background

The epidermal growth factor (EGF) receptor (EGFR) is a receptor tyrosine (Tyr) kinase which is activated via extracellular ligand binding and induces downstream signaling pathways that regulate cell proliferation, differentiation, migration, and survival [1, 2]. Mutation and/or amplification of the *EGFR* gene leading to aberrant activation of downstream signaling pathways are critical mechanisms promoting tumorigenesis, especially in lung cancer [3, 4]. Lung cancers are classified into small-cell lung cancer (SCLC) and non-SCLC (NSCLC). Pathologically, NSCLC accounts for 80% of lung cancer patients and is further categorized into adenocarcinomas, squamous cell carcinomas, and large-cell carcinomas based on the cytology [5]. Moreover, aberrant expression of the EGFR was found in 43%~ 86% of NSCLC patients [6]. Although gefitinib, an EGFR Tyr kinase inhibitor (TKI), was used to treat NSCLC patients 13 years ago [7], highly metastatic properties and drug resistance during TKI therapy still cause poor prognoses of lung cancer patients [8, 9]. Even now, the precise mechanism of resistance remains unclear, but it is well known that reactive oxygen species (ROS) are heavily involved in cancer initiation and regulation [10]. Exposure to environmental toxins, such as secondhand cigarette smoke and cooking smoke which are associated with oxidative stress formation, is an important mechanism participating in lung tumorigenesis through regulation of the EGFR-mediated signaling pathway [11, 12]. Therefore, understanding mechanisms between EGFR signaling pathways and oxidative stress-promoted lung tumorigenesis is necessary for lung cancer treatment and/or prevention.

Oxidative stress is defined as the overproduction of oxidants and/or a reduction in antioxidant defense abilities, which result in an imbalance of cellular oxidants and antioxidants [13]. Excessive production of oxidants comes from ROS/reactive nitrogen species (RNS) formation; these substances exhibit highly reactive activities toward cellular structural components, enzymes, and genetic materials leading to induction of inflammation, cell death, and tumorigenesis [14–16]. Furthermore, suppression of antioxidative enzyme expressions and activities by toxic materials also result in the emergence of oxidative stress. Basically, oxidative stress-regulated reduction-oxidation reaction (redox) signaling pathways are well characterized as risk factors for cancer progression [15]. Blockade of oxidative stress-mediated signaling pathways is a good strategy for cancer treatment and prevention [17, 18]. In this review, we summarized interactions of the EGFR and oxidative stress in tumor progression and TKI drug resistance.

### The role of the EGFR in tumor progression

The EGFR belongs to the HER family of transmembrane receptor Tyr kinases and consists of four related receptors: EGFR/HER1/ErbB1, HER2/ErbB2/Neu, HER3/ErbB3, and HER4/ErbB4 [19]. The EGFR is comprised of three domains: a glycosylated extracellular ligand-binding domain, a transmembrane domain, and an intercellular Tyr kinase domain. In normal physiological situations, EGFR activation is triggered by ligand binding and induction of EGFR homo- or heterodimerization. After receptor dimerization, Tyr residues in the Tyr kinase domain are autophosphorylated and activate downstream signaling pathways, such as Ras-Raf-extracellular signal-regulated kinase (ERK), phosphatidylinositol-3 kinase (PI3K)-Akt, and signal transducer and activator of transcription (STAT) signaling pathways, which regulate cell proliferation, migration, and survival [20]. However, aberrant expression of the EGFR via gene amplification, mutation, or protein overexpression results in dysregulation of EGFR-mediated signaling pathways with subsequent tumorigenesis, especially in lung cancer [21–23]. Several anticancer agents as EGFR Tyr kinase inhibitors (TKIs) have been used in NSCLC patients with EGFR mutations [24]. However, resistance to EGFR TKIs through an EGFR T790 M mutation, MET amplification, and/or activation of other kinases has limited the application of clinical anticancer agents for NSCLC treatment [25]. Therefore, understanding EGFR-regulated signaling pathways or cross-talk of other molecular mechanisms with EGFR signaling pathways may provide a way to treat or prevent lung cancer.

### The role of oxidative stress in tumor progression

“Oxidative stress” is defined as an imbalance between oxidant production and antioxidant defense systems. Overproduction of ROS/RNS by cellular metabolism or induction by exogenous sources triggers oxidative stress formation. ROS/RNS include free radicals and non-free radicals that have highly reactive activities with cellular components, such as lipids, proteins, and nucleic acids, and result in cellular dysfunction, gene mutations, tumor progression, and chemotherapeutic resistance [13, 26–29]. The majority of ROS/RNS are produced by mitochondrial metabolism, endoplasmic reticular stress, and peroxisomes. Reactions with intracellular enzymes, such as oxidase and oxygenase, also generate ROS/RNS [30, 31].

**Fig. 1:**
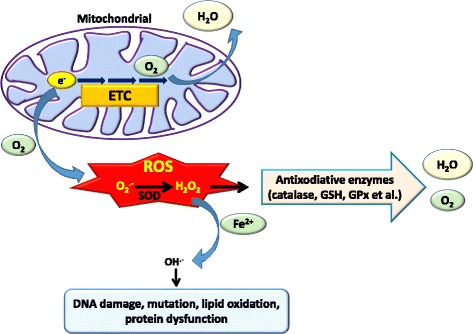
Generation of reactive oxygen species (ROS) from mitochondria. O_2_^·−^ is formed from molecular O_2_ by the gain of a single electron from electron leakage in the electron transport chain (ETC) of mitochondria. Superoxide dismutase (SOD) enzymes convert two superoxide molecules into hydrogen peroxide (H_2_O_2_) and a water (H_2_O) molecule. Moreover, hydrogen peroxide is converted to the highly reactive ROS, the hydroxyl radical (OH^.-^), in the presence of iron (Fe^2+^), which further causes damage to the cell structure including proteins, lipids, membranes, and DNA. Alternatively, hydrogen peroxide can be reduced to water by glutathione (GSH) peroxidase (GPx) or catalase

**Fig. 2:**
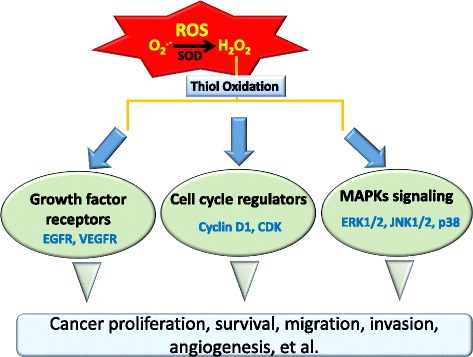
Schematic of possible mechanisms of reactive oxygen species (ROS)-induced tumor progression. Excessive intracellular ROS in cancer cells can regulate growth factor receptors, cell cycle regulators, and mitogen-activated protein kinase (MAPK) signaling that contribute to cancer progression by promoting cell proliferation, survival, migration, invasion, and angiogenesis

Mitochondrial ROS are metabolic byproducts and are produced from electron leakage during oxidative phosphorylation in the electron transport chain (ETC). Electrons escape from the ETC and bind to oxygen, with its subsequent reduction to superoxide anions (O_2_**·**^−^) and induction of oxidative stress [31]. Complex I (NADH-ubiquinone oxidase) and complex III (ubiquinone-cytochrome c oxidoreductase) of the ETC are major sites of superoxide anion generation. It was demonstrated that complex I is the primary site of superoxide anion production, and superoxide anions generated from complex III are an activator of hypoxia-inducible factor (HIF) in both pathological and physiological conditions [31–33]. Higher concentrations of superoxide anions are observed in cancer cells than in normal cells because of mitochondrial dysfunction [31]. The oxidative stress induced by superoxide anions leads to destruction of mitochondria and genomic DNA followed by gene mutations. Furthermore, superoxide anions also disturb signaling pathways and result in cancer proliferation, survival, and malignancy [34]. In addition, superoxide anions are further catalyzed to hydrogen peroxide (H_2_O_2_) by superoxide dismutase (SOD). Although hydrogen peroxide is not a free radical, it easily catalyzes free radical reactions and induces oxidative stress in living cells. For example, hydrogen peroxide can accept another electron from free Fe^2+^ by the Fenton reaction to become a hydroxyl radical (OH^.-^) and cause DNA damage [27, 35]. Hydrogen peroxide induces several types of DNA damage, including single- and double-stranded DNA breaks, DNA-protein cross links, and purine, pyrimidine, and deoxyribose modifications [36] (Fig. 1). Although changes in DNA are usually repaired by cells, persistent DNA damage causes replication errors, genomic instability, activation of oncogenes, and inactivation of tumor suppressor genes, and ultimately induces development of a variety of cancers [37]. Moreover, hydrogen peroxide induces oxidative stress and also regulates physiological signal transduction as a secondary messenger. ROS-mediated oxidation of cellular thiol-containing compounds, such as protein cysteine (Cys) residues, is considered to play important roles in a wide array of biological processes including signal transduction and regulation of the activities of enzymes, protein channels, and transcription factors [38]. For example, hydrogen peroxide was reported to modulate specific Cys residues within redox-sensitive proteins such as the EGFR [39–41]. Moreover, numerous signal molecules, such as the phosphatase and tensin homolog (PTEN), cyclin-dependent kinase (CDK), mitogen-activated protein kinases (MAPKs), and the transcription factor, activator protein (AP)-1, are also regulated by hydrogen peroxide [42–45] (Fig. 2).

### The role of ROS in tumor angiogenesis

**Fig. 3:**
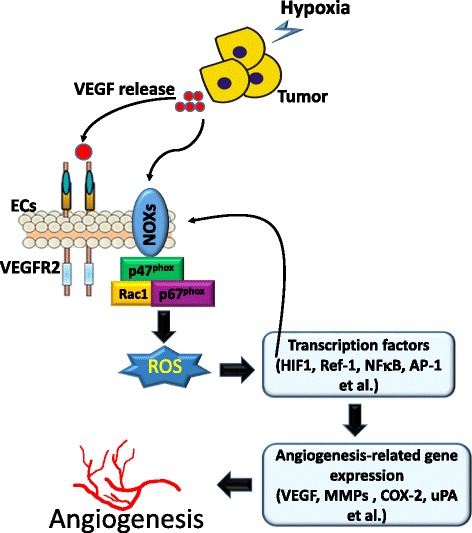
Roles of NADPH oxidase (NOX)-derived reactive oxygen species (ROS) in regulating angiogenesis. Hypoxia induces vascular endothelial growth factor (VEGF) production from tumor cells which stimulates NOXs to produce ROS, thereby inducing downstream redox signaling events including expressions of various redox-sensitive transcriptional factors (hypoxia-inducible factor (HIF)-1, redox factor (Ref)-1, nuclear factor (NF)-κB, and activator protein (AP)-1) and genes (VEGF, matrix metalloproteinases (MMPs), cyclooxygenase (COX)-2, and urokinase plasminogen activator (uPA)), which are involved in angiogenesis. ECs, endothelial cells

Angiogenesis is an essential step in tumorigenesis and metastasis. Until now, accumulating evidence suggested that ROS function as signaling molecules to mediate angiogenesis [46]. Numerous enzymatic systems capable of producing ROS were demonstrated, including cyclooxygenase (COX), nitric oxide synthase (NOS), the NADPH oxidase (NOX) family, etc. One of the major sources of ROS in endothelial cells (ECs) is NOX [46]. NOX family members are multiprotein complexes anchored to plasma membranes. Phagocyte oxidase was the first NOX identified, and it is required to defend against pathogenic infections of the respiratory system [47]. The NOX family has seven members, NOX1~ 5 and dual oxidase 1 and 2 (DUOX1 and − 2) [48]. Generally, NOX shows relatively low activity under physiologic conditions. High NOX activity was shown to correlate with ROS production and tumorigenesis and can be induced by chemicals, growth factors, and inflammatory factors [49, 50]. Different NOX members exhibit specific functions at both the tissue and cellular levels, and stimuli. For example, NOX1 is mainly expressed in the colon epithelium and is induced by angiotensin II [51], platelet-derived growth factor (PDGF) [52], and prostaglandins [53]. NOX4 has a wide tissue distribution, including ECs and the kidneys, and is activated by phorbol ester and insulin [54]. Moreover, expressions of NOX4 and NOX5 were shown to be higher in melanoma cells, prostate cancer cells, and lung cancer cells [55–57]. Recently, Han et al. further demonstrated that NOX activity and expression are associated with tumorigenesis of lung cancer, and inhibition of NOX function or messenger (m)RNA expression significantly blocks lung cancer formation and invasion [58]. Several NOX enzymes, including NOX1, NOX2, NOX4, and NOX5, as well as cytosolic regulatory subunits, including p47^phox^, p67^phox^, and Rac1, were shown to be involved in ROS production in ECs [46]. According to the type of ROS generated, NOX family members can be further divided into superoxide generators (NOX1~ 3 and NOX5) and hydrogen peroxide producers (NOX4 and DUOX1 and − 2) [48]. Previously, Arbiser et al. demonstrated that NOX-mediated upregulation of hydrogen peroxide induces vascular endothelial growth factor (VEGF) and its receptor (VEGFR-2) expressions and matrix metalloproteinase (MMP) activities, markers of the angiogenic switch, thereby promoting angiogenesis and expansion of tumors [59]. In addition, several other angiogenesis-related transcription factors and genes are also regulated by ROS. For example, HIF-1, redox factor (Ref)-1, nuclear factor (NF)-κB, and AP-1 are redox-sensitive transcription factors, and COX-2, plasminogen activator inhibitor (PAI)-1, and urokinase plasminogen activator (uPA) are redox-sensitive genes [46]. In contrast to ROS-induced angiogenesis-related transcription factors and gene expressions, NOX is also activated by numerous stimuli including the VEGF, EGF, angiopoietin (Ang)-1, urotensin (U)-II, shear stress, and hypoxia in ECs [60, 61]. VEGF can activate Rac1-depedent NOX to induce ROS production in ECs [62]. Furthermore, ROS-induced HIF expression shows positive feedback regulation on NOX expression in ECs [61] (Fig. 3).

### Interaction between the EGFR and oxidative stress in tumor progression

Twenty years ago, Gamou and Shimizu first identified the connection between hydrogen peroxide and the EGFR. They treated human squamous carcinoma NA cells with hydrogen peroxide (0~ 1 mM) and found that hydrogen peroxide enhanced EGFR phosphorylation. They found a discrepancy between EGF-induced EGFR activation and hydrogen peroxide-induced EGFR activation, in that hydrogen peroxide might preferentially induce EGFR Tyr phosphorylation, whereas EGF stimulation would trigger both serine/threonine (Ser/Thr) and Tyr receptor phosphorylation [63]. Inhibition of Cys-dependent protein Tyr phosphatases (PTPs) by hydrogen peroxide may be a critical mechanism inducing EGFR Tyr phosphorylation [64]. In addition, the intracellular kinase domain of the EGFR contains six Cys residues, and Cys797 is located in the ATP-binding pocket and is preferentially targeted by endogenous hydrogen peroxide [65]. Cys797 is a specific site of oxidation, and increased EGFR kinase activity after Cys797 oxidation was observed [66, 67]. Therefore, oxidation of specific residues in PTPs and/or the EGFR contribute to increased downstream signaling. Moreover, Goldkorn et al. also demonstrated that exogenous hydrogen peroxide stimulated EGFR Tyr kinase activity and increased the receptor half-life in NSCLC cells. They found that the half-life of the EGFR was about 8 h when treated with EGF, whereas hydrogen peroxide treatment prolonged the half-life of the EGFR to 18 h. According to those data, the authors suggested that EGF- and hydrogen peroxide-induced EGFR activation may have separate functions and represent an alternate mechanism by which EGFR signaling can be tuned in parallel to treatment with its native ligand [68].

**Fig. 4:**
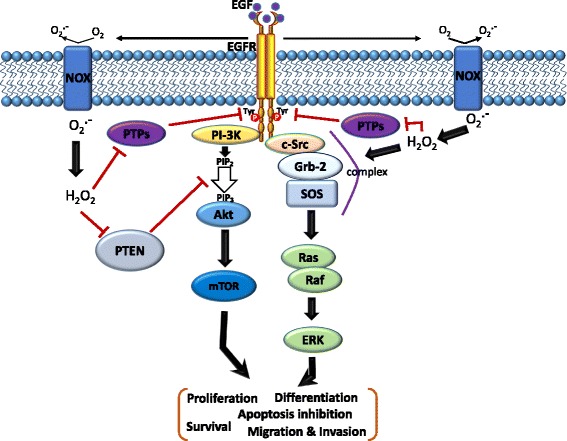
Schematic of the cross-talk between the epidermal growth factor (EGF)-EGF receptor (EGFR) axis and NADPH oxidase (NOX)-mediated reactive oxygen species (ROS) signaling pathways. The binding of EGF to the EGFR induces receptor dimerization and then autophosphorylation of tyrosine (Tyr) residues (red circles) in its cytoplasmic domain. These phosphorylated Tyr residues serve as docking sites for associated proteins which activate multiple pathways. In particular, the Ras/Raf/mitogen-activated protein kinase (MAPK) and phosphatidylinositol-3-kinase (PI3K)/Akt pathways downstream of the EGFR play critical roles in cell migration, invasion, proliferation, and survival. Moreover, the EGF-EGFR axis also induces NOX-mediated hydrogen peroxide production, and hydrogen peroxide can diffuse across the membrane to reach the intracellular cytosol. Transient increases in hydrogen peroxide induce oxidation of reduction-oxidation reaction (redox) targets such as phosphatase and tensin homolog (PTEN) to promote Akt activation, protein tyrosine (Tyr) phosphatases (PTPs) to enhance EGFR Tyr phosphorylation, or complex formation of SHC-Grb2-SOS with the EGFR to activate Ras/MAPK signaling. Grb-2, growth factor receptor-bound protein 2; SOS, guanine nucleotide exchange protein

In addition to the overall increase in Tyr autophosphorylation by the EGFR, hydrogen peroxide can also be a secondary messenger to regulate signal transduction [69]. After EGF stimulation, the EGFR transmits activation signals to downstream signaling cascades, including the Ras/MAPK and PI3K/Akt pathways. Hydrogen peroxide modulates the Src homology 2 domain-containing (SHC) protein, growth factor receptor-bound protein 2 (Grb2), and guanine nucleotide exchange protein (SOS) to form the SHC-Grb2-SOS complex [70, 71]. Once the SHC-Grb2-SOS complex is associated with the EGFR, the SOS promotes the exchange of GDP for GTP on Ras, a small G protein. Complexes that contain the SHC protein were reported to be better at activating RAS than those without it [72]. GTP-bound active Ras can then bind with and activate the MAPK kinase kinase (MAPKKK) protein, Raf. Raf can further induce the phosphorylation of a Ser residue in the activation loop of MEK (MAPKK) [73]. Thereafter, activated MEK1/2 phosphorylates the MAPK protein, extracellular signal-regulated kinase (ERK), on adjacent Thr and Tyr residues, separated by a glutamic acid residue, at the activation loop. Eventually, this pathway controls various cellular processes of cancer including growth, proliferation, differentiation, migration, and inhibition of apoptosis [74, 75] (Fig. 4).

The PI3K-Akt-mammalian target of rapamycin (mTOR) signaling cascade is a signal transduction pathway involved in regulating multiple cellular functions including proliferation, survival, cell size, migration, and invasion. In cancer, this pathway is often hyperactivated due to activating mutations of EGFR family members. Activation of the EGFR leads to recruitment and activation of Akt at plasma membranes by activating the PI3K-induced formation of phosphatidylinositol-3,4,5-triphosphate (PIP_3_) [76]. The signaling potential of PIP_3_ is controlled by the PI3K antagonist, PTEN, which dephosphorylates and limits the activity of PIP_3_ and is frequently inactivated in cancer [77]. In addition, it was found that increased endogenous hydrogen peroxide through overexpression of NOX1 elevates intracellular PIP_3_ concentrations and promotes the PI3K/Akt signaling pathway due to inhibition of PTEN activity [78, 79]. Hydrogen peroxide inhibits phosphatase activity by triggering the formation of a disulfide bond between the catalytic Cys, Cys124, and the closely aligned Cys, Cys71 [80] (Fig. 4). Akt is a Ser-Thr protein kinase which exists in three isoforms, including ubiquitously expressed Akt1 and Akt2, and Akt3, which is predominantly expressed in the brain, kidneys, and heart [81]. Similar to PTEN, Akt2 can form an intracellular disulfide bond between two Cys molecules in the activation loop, which inhibits kinase activity [82]. Elimination of hydrogen peroxide by overexpression of antioxidant enzymes, such as glutaredoxin, sustains Akt phosphorylation and inhibition of apoptosis through protecting Akt against hydrogen peroxide-induced oxidation [83].

In contrast to hydrogen peroxide-induced EGFR activation, the addition of EGF to human epidermoid carcinoma A431 cells, which highly express the EGFR, leads to increased levels of intracellular ROS, especially hydrogen peroxide [64] (Fig. 4). Moreover, EGF-induced Tyr phosphorylation of phospholipase Cγ1 (PLCγ1), a well-known physiological substrate of the EGFR, is inhibited through elimination of ROS by catalase, suggesting that inhibition of Cys-dependent PTPs by hydrogen peroxide might be required to increase the Tyr phosphorylation of PLCγ1 [64]. Until now, the precise molecular mechanisms of EGF-induced hydrogen peroxide generation are still not very clear.

### The role of ROS in drug resistance of EGFR TKIs

Targeting the EGFR is a strategy for lung cancer treatment and prevention. However, drug resistance has emerged after chronic treatment with EGFR TKIs. It was reported that high basal ROS levels were observed in TKI-resistant NSCLC cell lines, and high NOX2 expression-mediated ROS production was associated with poor patient survival in clinical lung tumors [11]. A previous report indicated that oxidation of Cys797 of the EGFR could promote binding of the EGFR to NOX2 and further enhance ROS generation and EGFR activation [40, 66]. Moreover, EGFR-sensitive NSCLC cell lines exposed to ROS resulted in TKI resistance by abnormal EGFR phosphorylation and disruption of the canonical dimer structure of the EGFR [84, 85]. Furthermore, cigarette smoke extract was reported to induce EGFR TKI resistance via promoting ROS generation and EGFR signaling pathway activation [85, 86]. In addition to NSCLC, ROS were also reported to play a critical role in mediating EGFR-TKI resistance in breast cancer cells [87]. Treatment of lung cancer cells with an EGFR-TKI was reported to increase ROS concentrations or Src activation and further induce epithelial-to-mesenchymal transition (EMT)-mediated drug resistance [88, 89]. The EMT was reported to play a critical role in cancer metastasis and EGFR-TKI resistance [90]. Src activation was indicated to regulate proliferation and the EMT in lung cancer [91]. Moreover, Src is one of the major downstream target molecules of ROS [92]. Taken together, interactions among ROS, Src, EGFR signaling, and the EMT are very important to EGFR-TKI resistance and might serve as druggable targets to overcome resistance to EGFR-TKIs. For example, combination treatment with TKIs and ROS scavengers partially reversed the EMT and restored mitochondrial functions [93, 94]. Accordingly, antioxidants could potentially provide therapeutic benefits by attenuating ROS-mediated tumor progression and TKI resistance. Although it is understood that antioxidants are beneficial in preventing NSCLC and other cancers [95–97], several clinical trials recently reported that dietary supplementation with antioxidants such as vitamin A, vitamin C, vitamin E, retinol, and beta-carotene did not significantly influence [98, 99] or further heightened [100–102] the risk of certain cancer types such as prostate, pancreas, head and neck, and lung cancers. The contradictory effects of antioxidants on cancers might be due to levels of ROS production by tumor cells.

### Low and high ROS concentrations play contrary roles in tumor progression and drug resistance

**Fig. 5:**
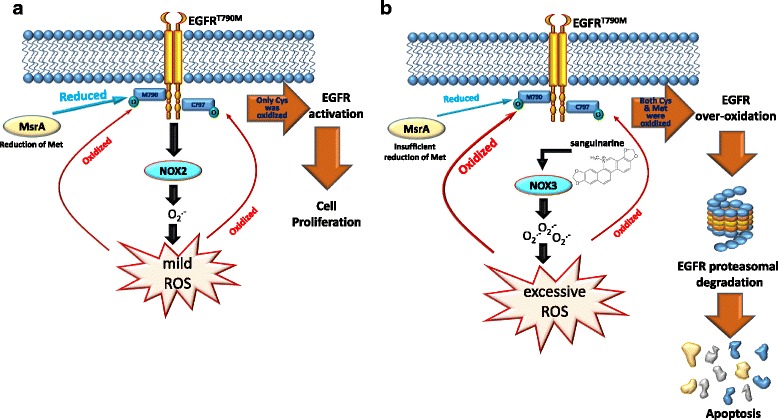
Different fates of epidermal growth factor (EGF)-EGF receptor (EGFR) harboring the T790 M mutation in basal and excessive reactive oxygen species (ROS) conditions. **a** In the basal condition, the EGFR T790 M mutant activates NADPH oxidase 2 (NOX2) to produce ROS, and further oxidizes cysteine (Cys)797 of the EGFR, resulting in mild oxidation of methionine (Met)790. In this condition, basal activity of Met reductase A (MsrA) is fully responsible for reducing the oxidized form of Met790 on the EGFR to the reduced form and protects it from degradation, resulting in cell survival. **b** Under a condition of sanguinarine-mediated ROS overproduction, a reduction-oxidation reaction (redox) imbalance is induced by activating NOX3 to cause oxidation and depletion of NADPH, resulting in MsrA inactivation and loss of the ability to reduce the oxidized form of Met790 on the EGFR. Excessive amounts of ROS can further induce overoxidation of Met790 on the EGFR and ultimately induce EGFR degradation and cell apoptosis

Chronic exposure to low ROS levels can effectively regulate mitosis, cell survival, cell growth, cell proliferation, and angiogenesis in cancers [103] Moreover, tumor-initiating cells (TICs) play an important role responsible for TKI and chemotherapeutic resistance in tumors [104], which maintain lower ROS levels than their more-mature progeny owing to expressions of ROS-scavenging molecules and mechanisms such as CD13, glutathione synthetase, glutamate cysteine ligase, nuclear factor-erythroid 2-related factor 2 (NRF2), and Forkhead box protein O1 (FOXO1)-mediated upregulation of SOD and catalase [17, 105–107]. Overexpression of drug efflux pumps in TICs is an important mechanism for drug resistance of TICs. Low concentrations of ROS in TICs were reported to induce upregulation of the drug efflux pump, P-glycoprotein (P-gp), which promoted acquisition of the multidrug resistance (MDR) phenotype [108, 109]. Overexpression of P-gp was found with a low concentration (1 μM) of hydrogen peroxide treatment; however, a high concentration (10 μM) of hydrogen peroxide treatment suppressed the expression of P-gp in cells, hence producing a concentration-dependent contribution of ROS to P-gp-mediated drug resistance [110]. Patients with TKI-resistant NSCLC and an additional EGFR^T790M^ mutation represent almost half of all NSCLC TKI-resistant cases [111]. In a basal condition of H1975 NSCLC cells harboring the EGFR^T790M^ mutant, activated EGFR^T790M^ can induce NOX2-mediated upregulation of ROS, further oxidizing Cys797 and methionine (Met)790 of the EGFR. Mild oxidation of Met790 can be physiologically reversed to the reduced form by the Met reductase, Met reductase A (MsrA), and oxidation of Cys797 on EGFR^T790M^ can further induce EGFR activation and cell proliferation [11, 66] (Fig. 5a). In contrast to low ROS levels, high ROS levels were shown to have toxic effects on cancer cells, by triggering several signal transduction pathways to induce cell cycle arrest and cell death and overcome TKI resistance [11, 15]. For example, apoptosis signal-regulating kinase 1 (ASK1) acts as a redox sensor by activating JNK and p38 MAPK to induce apoptosis upon excessive ROS [112]. ROS-induced p38/JNK MAPK signaling can downregulate cyclins and induce CDK inhibitors resulting in cell cycle arrest [113]. In EGFR-TKI-resistant H1975 cells, Leung et al. indicated that sanguinarine, a powerful ROS producer, caused excessive accumulation of ROS by activating NOX3 to further inactivate MsrA and induce overoxidation of EGFR^T790M^, and finally mediate EGFR degradation and cell death. This finding indicated that induction of NOX3 activation and Met790 oxidation is a novel target for treating NSCLC patients with the EGFR^T790M^ mutation [11] (Fig. 5b). Moreover, overproduction of ROS through the simultaneous induction of superoxide and hydrogen peroxide was reported to synergistically induce the death of NSCLC cells [114]. Taken together, the information detailed above indicates that maintaining endogenous and induced ROS at moderate levels can mediate drug resistance and allow tumor cells to survive treatment, resulting in both stemness and cancer-initiating capabilities.

## Summary and concluding remarks

An imbalance of oxidants and antioxidants decides ROS levels in tissues. ROS-induced oxidative stress is a double-edged sword in cancers. Moderate ROS levels play positive roles in cancer such as promoting inflammation, tumor progression, and drug resistance. In contrast, excessive ROS can induce negative responses such as growth inhibition or death of cancer cells [115]. Mitochondrial dysfunction is the major mechanism inducing oxidative stress. Furthermore, dysregulation of enzymatic reactions also produces ROS in cancer cells, such as NOX. Reversing mitochondrial function and antioxidative enzyme activity can suppress ROS-mediated tumor progression. ROS not only function as mediators of the EGF/EGFR signaling pathway, but also as regulators of the oxidation status and activation of the EGFR protein. Oxidation of PTPs and/or specific Cys residues of the EGFR induce the EGFR-regulated signaling pathway by mild levels of ROS [64, 67]. On the contrary, higher ROS levels can trigger overoxidation of the Met residue of EGFR^T790M^ and shut down the EGFR downstream survival pathway [11]. Therefore, modulating ROS levels in cancers may be a feasible therapeutic approach for cancer treatment and prevention. Although there are some studies which show that antioxidants can be used for cancer treatment [116, 117], recent reports indicate that using antioxidants in clinical trials might be associated with increased cancer incidences and a reduced efficacy of chemotherapy [115]. According to recent research, inhibiting metabolic pathways or ROS-scavenging mechanisms in the tumor itself, followed by treatment with pro-oxidizing agents such as chemotherapeutic drugs, represents alternative and promising therapeutic options for tumors characterized by resistance to treatment [118]. However, there are also some undesired side effects including cardiotoxicity and nephrotoxicity associated with this therapeutic strategy, and novel ROS modulators with a safer therapeutic profile should be further developed.

## Abbreviations

Ang: angiopoietin
ASK1: apoptosis signal-regulating kinase 1
CDK: cyclin-dependent kinase
COX: cyclooxygenase
Cys: cysteine
DUOX: dual oxidase
EC: endothelial cell
EGFR: epidermal growth factor receptor
ERK: extracellular signal-regulated kinase
ETC: electron transport chain
FOXO1: Forkhead box protein O1
Grb2: growth factor receptor-bound protein 2
HIF: hypoxia-inducible factor
MAPK: mitogen-activated protein kinase
MDR: multidrug resistance
Met: methionine
MMP: matrix metalloproteinase
MsrA: methionine reductase A
mTOR: mammalian target of rapamycin
NOS: nitric oxide synthase
NOX: NADPH oxidase
NRF2: nuclear factor-erythroid 2-related factor 2
NSCLC: non-small cell lung cancer
PAI: plasminogen activator inhibitor
PDGF: platelet-derived growth factor
P-gp: P-glycoprotein
PI3K: phosphatidylinositol-3 kinase
PTEN: phosphatase and tensin homolog
PTP: protein tyrosine phosphatase
Ref: redox factor
RNS: reactive nitrogen species
ROS: reactive oxygen species
Ser: serine
SHC: Src homology 2 domain-containing
SOD: superoxide dismutase
STAT: signal transducer and activator of transcription
Thr: threonine
TIC: tumor-initiating cell
TKI: tyrosine kinase inhibitor
uPA: urokinase plasminogen activator
VEGF: vascular endothelial growth factor
